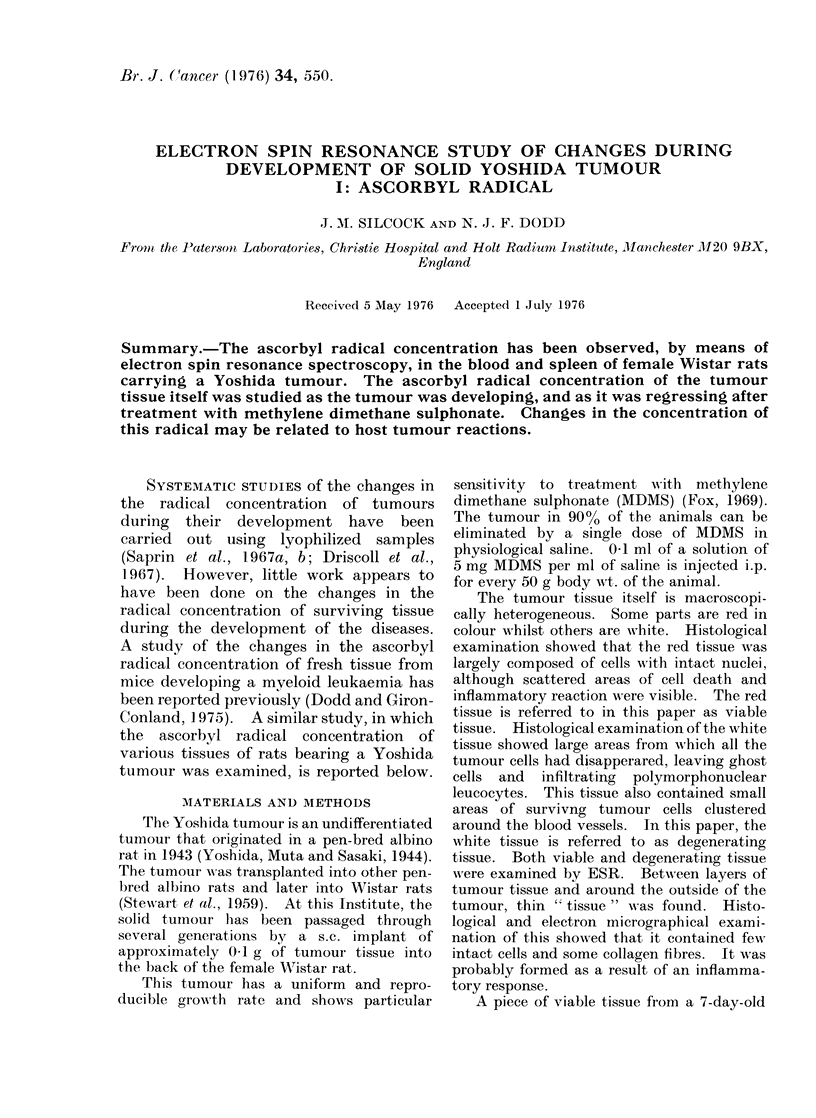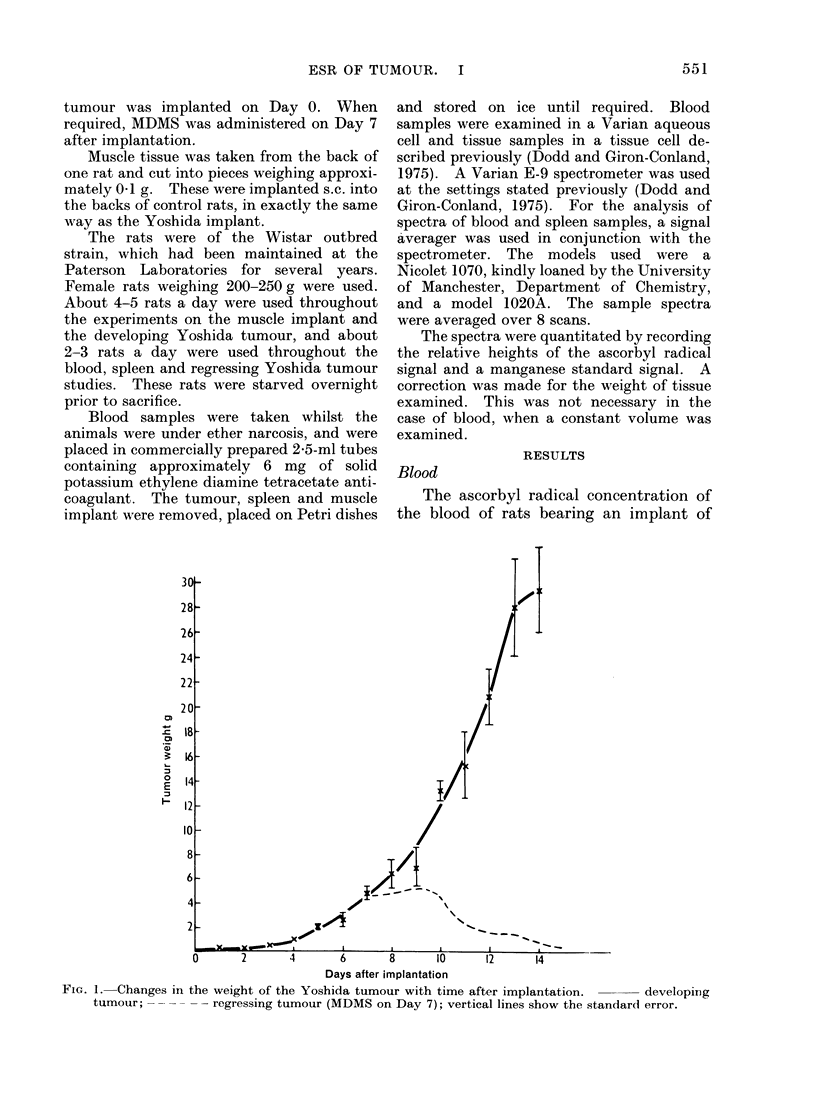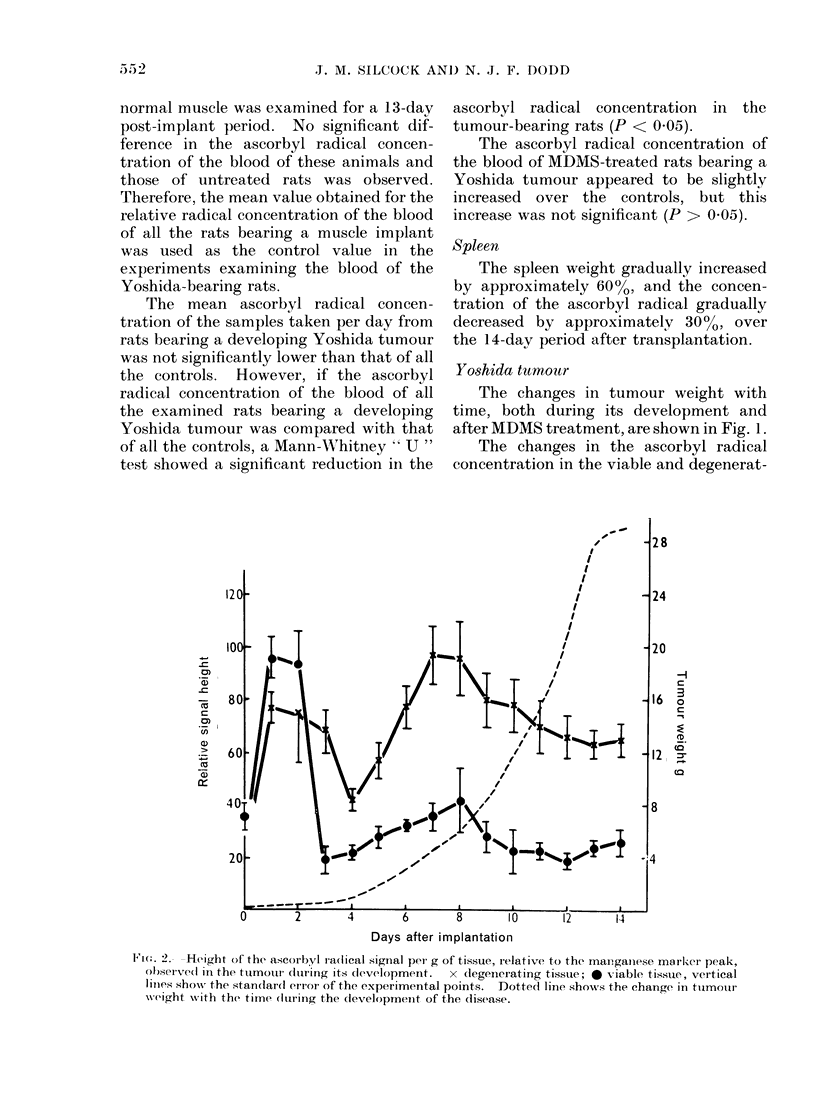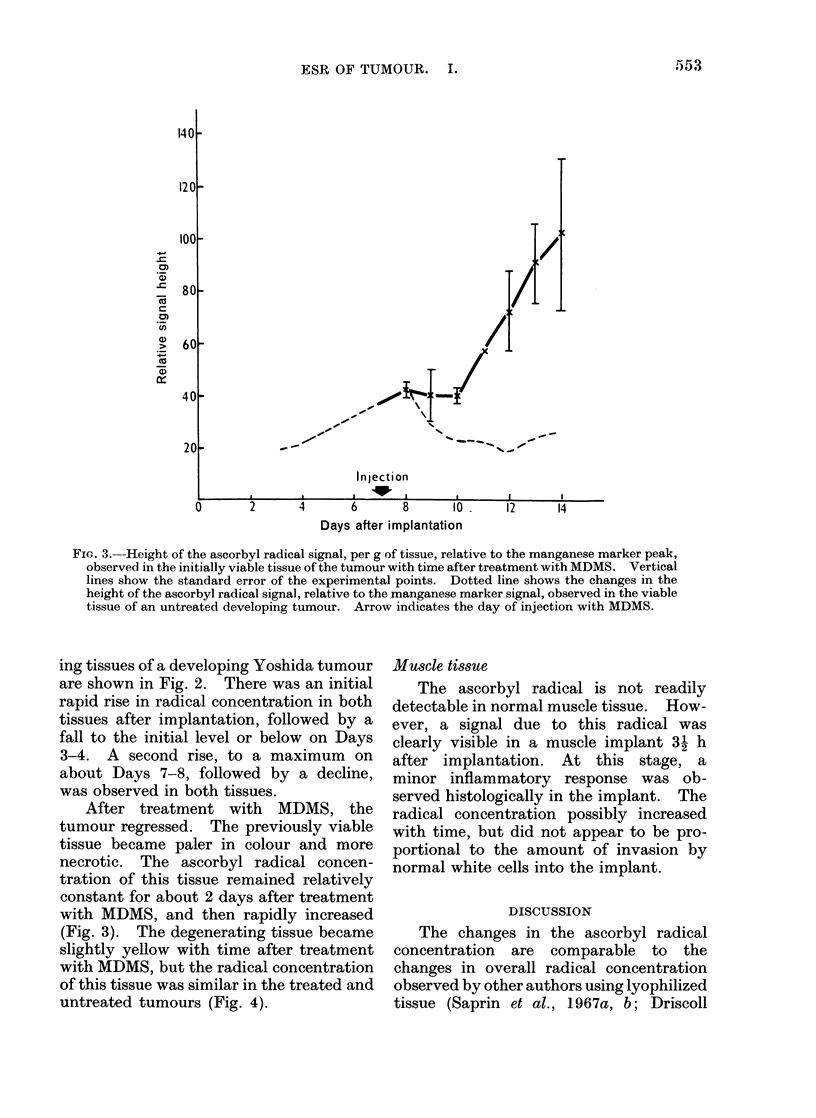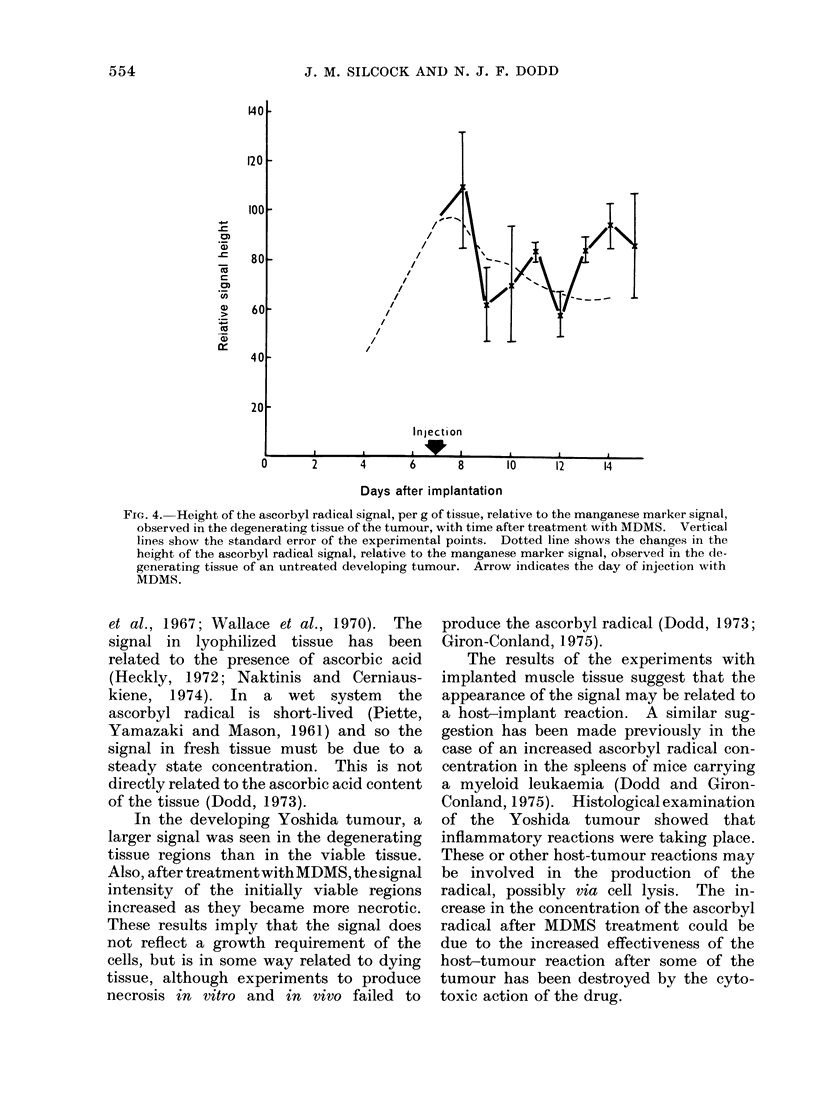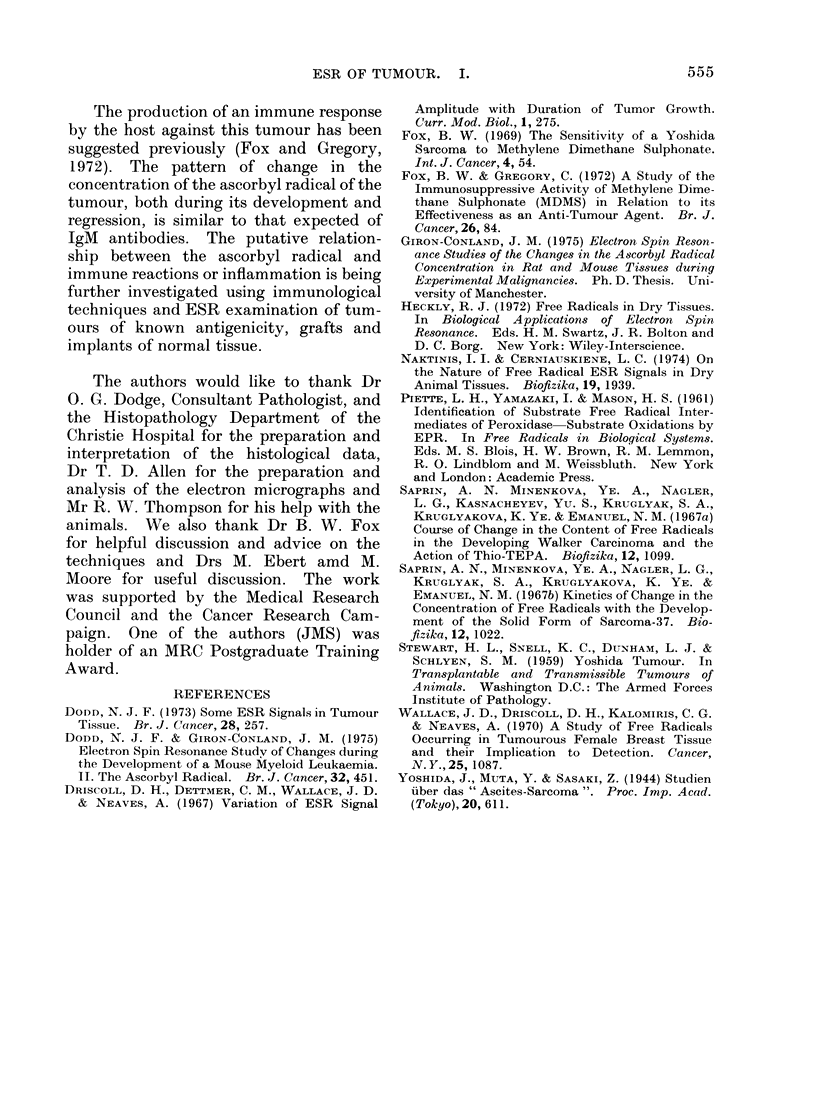# Electron spin resonance study of changes during development of solid Yoshida tumour. I: Ascorbyl radical.

**DOI:** 10.1038/bjc.1976.210

**Published:** 1976-11

**Authors:** J. M. Silcock, N. J. Dodd

## Abstract

The ascorbyl radical concentration has been observed, by means of electron spin resonance spectroscopy, in the blood and spleen of female Wistar rats carrying a Yoshida tumour. The ascorbyl radical concentration of the tumour tissue itself was studied as the tumour was developing, and as it was regressing after treatment with methylene dimethane sulphonate. Changes in the concentration of this radical may be related to host tumour reactions.


*Br. J. Cancer* (1976) **34**, 550.

### ELECTRON SPIN RESONANCE STUDY OF CHANGES DURING DEVELOPMENT OF SOLID YOSHIDA TUMOUR I: ASCORBYL RADICAL

J. M. SILCOCK AND N. J. F. DODD

*From the Paterson Laboratories, Christie Hospital and Holt Radium Institute, Manchester M20 9BX, England*

Received 5 May 1976 Accepted 1 July 1976

**Summary.—The ascorbyl radical concentration has been observed, by means of electron spin resonance spectroscopy, in the blood and spleen of female Wistar rats carrying a Yoshida tumour. The ascorbyl radical concentration of the tumour tissue itself was studied as the tumour was developing, and as it was regressing after treatment with methylene dimethane sulphonate. Changes in the concentration of this radical may be related to host tumour reactions.**

SYSTEMATIC STUDIES of the changes in the radical concentration of tumours during their development have been carried out using lyophilized samples (Saprin *et al.*, 1967*a*, *b*; Driscoll *et al.*, 1967). However, little work appears to have been done on the changes in the radical concentration of surviving tissue during the development of the diseases. A study of the changes in the ascorbyl radical concentration of fresh tissue from mice developing a myeloid leukaemia has been reported previously (Dodd and Giron-Conland, 1975). A similar study, in which the ascorbyl radical concentration of various tissues of rats bearing a Yoshida tumour was examined, is reported below.

### MATERIALS AND METHODS

The Yoshida tumour is an undifferentiated tumour that originated in a pen-bred albino rat in 1943 (Yoshida, Muta and Sasaki, 1944). The tumour was transplanted into other pen-bred albino rats and later into Wistar rats (Stewart *et al.*, 1959). At this Institute, the solid tumour has been passaged through several generations by a s.c. implant of approximately 0·1 g of tumour tissue into the back of the female Wistar rat.

This tumour has a uniform and reproducible growth rate and shows particular sensitivity to treatment with methylene dimethane sulphonate (MDMS) (Fox, 1969). The tumour in 90% of the animals can be eliminated by a single dose of MDMS in physiological saline. 0·1 ml of a solution of 5 mg MDMS per ml of saline is injected i.p. for every 50 g body wt. of the animal.

The tumour tissue itself is macroscopically heterogeneous. Some parts are red in colour whilst others are white. Histological examination showed that the red tissue was largely composed of cells with intact nuclei, although scattered areas of cell death and inflammatory reaction were visible. The red tissue is referred to in this paper as viable tissue. Histological examination of the white tissue showed large areas from which all the tumour cells had disapperared, leaving ghost cells and infiltrating polymorphonuclear leucocytes. This tissue also contained small areas of survivng tumour cells clustered around the blood vessels. In this paper, the white tissue is referred to as degenerating tissue. Both viable and degenerating tissue were examined by ESR. Between layers of tumour tissue and around the outside of the tumour, thin " tissue " was found. Histological and electron micrographical examination of this showed that it contained few intact cells and some collagen fibres. It was probably formed as a result of an inflammatory response.

A piece of viable tissue from a 7-day-old tumour was implanted on Day 0. When required, MDMS was administered on Day 7 after implantation.

Muscle tissue was taken from the back of one rat and cut into pieces weighing approximately 0·1 g. These were implanted s.c. into the backs of control rats, in exactly the same way as the Yoshida implant.

The rats were of the Wistar outbred strain, which had been maintained at the Paterson Laboratories for several years. Female rats weighing 200–250 g were used. About 4–5 rats a day were used throughout the experiments on the muscle implant and the developing Yoshida tumour, and about 2–3 rats a day were used throughout the blood, spleen and regressing Yoshida tumour studies. These rats were starved overnight prior to sacrifice.

Blood samples were taken whilst the animals were under ether narcosis, and were placed in commercially prepared 2·5-ml tubes containing approximately 6 mg of solid potassium ethylene diamine tetracetate anticoagulant. The tumour, spleen and muscle implant were removed, placed on Petri dishes and stored on ice until required. Blood samples were examined in a Varian aqueous cell and tissue samples in a tissue cell described previously (Dodd and Giron-Conland, 1975). A Varian E-9 spectrometer was used at the settings stated previously (Dodd and Giron-Conland, 1975). For the analysis of spectra of blood and spleen samples, a signal averager was used in conjunction with the spectrometer. The models used were a Nicolet 1070, kindly loaned by the University of Manchester, Department of Chemistry, and a model 1020A. The sample spectra were averaged over 8 scans.

The spectra were quantitated by recording the relative heights of the ascorbyl radical signal and a manganese standard signal. A correction was made for the weight of tissue examined. This was not necessary in the case of blood, when a constant volume was examined.

### RESULTS

### *Blood*

The ascorbyl radical concentration of the blood of rats bearing an implant of

FIG. 1.—Changes in the weight of the Yoshida tumour with time after implantation. ——— developing tumour; – – – – – – regressing tumour (MDMS on Day 7); vertical lines show the standard error.

normal muscle was examined for a 13-day post-implant period. No significant difference in the ascorbyl radical concentration of the blood of these animals and those of untreated rats was observed. Therefore, the mean value obtained for the relative radical concentration of the blood of all the rats bearing a muscle implant was used as the control value in the experiments examining the blood of the Yoshida-bearing rats.

The mean ascorbyl radical concentration of the samples taken per day from rats bearing a developing Yoshida tumour was not significantly lower than that of all the controls. However, if the ascorbyl radical concentration of the blood of all the examined rats bearing a developing Yoshida tumour was compared with that of all the controls, a Mann-Whitney " U " test showed a significant reduction in the ascorbyl radical concentration in the tumour-bearing rats ($P < 0{\cdot}05$).

The ascorbyl radical concentration of the blood of MDMS-treated rats bearing a Yoshida tumour appeared to be slightly increased over the controls, but this increase was not significant ($P > 0{\cdot}05$).

*Spleen*

The spleen weight gradually increased by approximately 60%, and the concentration of the ascorbyl radical gradually decreased by approximately 30%, over the 14-day period after transplantation.

*Yoshida tumour*

The changes in tumour weight with time, both during its development and after MDMS treatment, are shown in Fig. 1.

The changes in the ascorbyl radical concentration in the viable and degenerat-

FIG. 2.—Height of the ascorbyl radical signal per g of tissue, relative to the manganese marker peak, observed in the tumour during its development. × degenerating tissue; ● viable tissue, vertical lines show the standard error of the experimental points. Dotted line shows the change in tumour weight with the time during the development of the disease.

FIG. 3.—Height of the ascorbyl radical signal, per g of tissue, relative to the manganese marker peak, observed in the initially viable tissue of the tumour with time after treatment with MDMS. Vertical lines show the standard error of the experimental points. Dotted line shows the changes in the height of the ascorbyl radical signal, relative to the manganese marker signal, observed in the viable tissue of an untreated developing tumour. Arrow indicates the day of injection with MDMS.

ing tissues of a developing Yoshida tumour are shown in Fig. 2. There was an initial rapid rise in radical concentration in both tissues after implantation, followed by a fall to the initial level or below on Days 3–4. A second rise, to a maximum on about Days 7–8, followed by a decline, was observed in both tissues.

After treatment with MDMS, the tumour regressed. The previously viable tissue became paler in colour and more necrotic. The ascorbyl radical concentration of this tissue remained relatively constant for about 2 days after treatment with MDMS, and then rapidly increased (Fig. 3). The degenerating tissue became slightly yellow with time after treatment with MDMS, but the radical concentration of this tissue was similar in the treated and untreated tumours (Fig. 4).

*Muscle tissue*

The ascorbyl radical is not readily detectable in normal muscle tissue. However, a signal due to this radical was clearly visible in a muscle implant $3\frac{1}{2}$ h after implantation. At this stage, a minor inflammatory response was observed histologically in the implant. The radical concentration possibly increased with time, but did not appear to be proportional to the amount of invasion by normal white cells into the implant.

### DISCUSSION

The changes in the ascorbyl radical concentration are comparable to the changes in overall radical concentration observed by other authors using lyophilized tissue (Saprin *et al.*, 1967*a*, *b*; Driscoll

FIG. 4.—Height of the ascorbyl radical signal, per g of tissue, relative to the manganese marker signal, observed in the degenerating tissue of the tumour, with time after treatment with MDMS. Vertical lines show the standard error of the experimental points. Dotted line shows the changes in the height of the ascorbyl radical signal, relative to the manganese marker signal, observed in the degenerating tissue of an untreated developing tumour. Arrow indicates the day of injection with MDMS.

*et al.*, 1967; Wallace *et al.*, 1970). The signal in lyophilized tissue has been related to the presence of ascorbic acid (Heckly, 1972; Naktinis and Cerniauskiene, 1974). In a wet system the ascorbyl radical is short-lived (Piette, Yamazaki and Mason, 1961) and so the signal in fresh tissue must be due to a steady state concentration. This is not directly related to the ascorbic acid content of the tissue (Dodd, 1973).

In the developing Yoshida tumour, a larger signal was seen in the degenerating tissue regions than in the viable tissue. Also, after treatment with MDMS, the signal intensity of the initially viable regions increased as they became more necrotic. These results imply that the signal does not reflect a growth requirement of the cells, but is in some way related to dying tissue, although experiments to produce necrosis *in vitro* and *in vivo* failed to produce the ascorbyl radical (Dodd, 1973; Giron-Conland, 1975).

The results of the experiments with implanted muscle tissue suggest that the appearance of the signal may be related to a host–implant reaction. A similar suggestion has been made previously in the case of an increased ascorbyl radical concentration in the spleens of mice carrying a myeloid leukaemia (Dodd and Giron-Conland, 1975). Histological examination of the Yoshida tumour showed that inflammatory reactions were taking place. These or other host-tumour reactions may be involved in the production of the radical, possibly *via* cell lysis. The increase in the concentration of the ascorbyl radical after MDMS treatment could be due to the increased effectiveness of the host–tumour reaction after some of the tumour has been destroyed by the cytotoxic action of the drug.

The production of an immune response by the host against this tumour has been suggested previously (Fox and Gregory, 1972). The pattern of change in the concentration of the ascorbyl radical of the tumour, both during its development and regression, is similar to that expected of IgM antibodies. The putative relationship between the ascorbyl radical and immune reactions or inflammation is being further investigated using immunological techniques and ESR examination of tumours of known antigenicity, grafts and implants of normal tissue.

The authors would like to thank Dr O. G. Dodge, Consultant Pathologist, and the Histopathology Department of the Christie Hospital for the preparation and interpretation of the histological data, Dr T. D. Allen for the preparation and analysis of the electron micrographs and Mr R. W. Thompson for his help with the animals. We also thank Dr B. W. Fox for helpful discussion and advice on the techniques and Drs M. Ebert amd M. Moore for useful discussion. The work was supported by the Medical Research Council and the Cancer Research Campaign. One of the authors (JMS) was holder of an MRC Postgraduate Training Award.

### REFERENCES

DODD, N. J. F. (1973) Some ESR Signals in Tumour Tissue. *Br. J. Cancer*, **28**, 257.

DODD, N. J. F. & GIRON-CONLAND, J. M. (1975) Electron Spin Resonance Study of Changes during the Development of a Mouse Myeloid Leukaemia. II. The Ascorbyl Radical. *Br. J. Cancer*, **32**, 451.

DRISCOLL, D. H., DETTMER, C. M., WALLACE, J. D. & NEAVES, A. (1967) Variation of ESR Signal Amplitude with Duration of Tumor Growth. *Curr. Mod. Biol.*, **1**, 275.

FOX, B. W. (1969) The Sensitivity of a Yoshida Sarcoma to Methylene Dimethane Sulphonate. *Int. J. Cancer*, **4**, 54.

FOX, B. W. & GREGORY, C. (1972) A Study of the Immunosuppressive Activity of Methylene Dimethane Sulphonate (MDMS) in Relation to its Effectiveness as an Anti-Tumour Agent. *Br. J. Cancer*, **26**, 84.

GIRON-CONLAND, J. M. (1975) *Electron Spin Resonance Studies of the Changes in the Ascorbyl Radical Concentration in Rat and Mouse Tissues during Experimental Malignancies.* Ph. D. Thesis. University of Manchester.

HECKLY, R. J. (1972) Free Radicals in Dry Tissues. In *Biological Applications of Electron Spin Resonance.* Eds. H. M. Swartz, J. R. Bolton and D. C. Borg. New York: Wiley-Interscience.

NAKTINIS, I. I. & CERNIAUSKIENE, L. C. (1974) On the Nature of Free Radical ESR Signals in Dry Animal Tissues. *Biofizika*, **19**, 1939.

PIETTE, L. H., YAMAZAKI, I. & MASON, H. S. (1961) Identification of Substrate Free Radical Intermediates of Peroxidase—Substrate Oxidations by EPR. In *Free Radicals in Biological Systems.* Eds. M. S. Blois, H. W. Brown, R. M. Lemmon, R. O. Lindblom and M. Weissbluth. New York and London: Academic Press.

SAPRIN, A. N. MINENKOVA, YE. A., NAGLER, L. G., KASNACHEYEV, YU. S., KRUGLYAK, S. A., KRUGLYAKOVA, K. YE. & EMANUEL, N. M. (1967*a*) Course of Change in the Content of Free Radicals in the Developing Walker Carcinoma and the Action of Thio-TEPA. *Biofizika*, **12**, 1099.

SAPRIN, A. N., MINENKOVA, YE. A., NAGLER, L. G., KRUGLYAK, S. A., KRUGLYAKOVA, K. YE. & EMANUEL, N. M. (1967*b*) Kinetics of Change in the Concentration of Free Radicals with the Development of the Solid Form of Sarcoma-37. *Biofizika*, **12**, 1022.

STEWART, H. L., SNELL, K. C., DUNHAM, L. J. & SCHLYEN, S. M. (1959) Yoshida Tumour. In *Transplantable and Transmissible Tumours of Animals.* Washington D.C.: The Armed Forces Institute of Pathology.

WALLACE, J. D., DRISCOLL, D. H., KALOMIRIS, C. G. & NEAVES, A. (1970) A Study of Free Radicals Occurring in Tumourous Female Breast Tissue and their Implication to Detection. *Cancer, N.Y.*, **25**, 1087.

YOSHIDA, J., MUTA, Y. & SASAKI, Z. (1944) Studien über das " Ascites-Sarcoma ". *Proc. Imp. Acad. (Tokyo)*, **20**, 611.